# Lipoprotein Lipase Genetic Variants rs258 and rs326 Differentially Affect Lipid Profiles and Leptin Levels in Prepubertal Spanish Caucasian Children

**DOI:** 10.3390/jcm15020493

**Published:** 2026-01-08

**Authors:** Olga Pomares, Iris Pérez-Nadador, Francisco J. Mejorado-Molano, Alejandro Parra-Rodríguez, Leandro Soriano-Guillén, Carmen Garcés

**Affiliations:** 1 Lipid Research Laboratory, Instituto de Investigación Sanitaria Fundación Jiménez Díaz (IIS-FJD, UAM), 28040 Madrid, Spain; olga.pomares@iis-fjd.es (O.P.); iris.perezn@iis-fjd.es (I.P.-N.); 2 Department of Pediatrics, Instituto de Investigación Sanitaria Fundación Jiménez Díaz (IIS-FJD, UAM), 28040 Madrid, Spain; fmejorado@quironsalud.es (F.J.M.-M.); alejandro.parra@quironsalud.es (A.P.-R.); lsoriano@fjd.es (L.S.-G.)

**Keywords:** Apo-B, LDL-cholesterol, leptin, LPL variants, prepubertal children

## Abstract

**Background/Objectives:** Variants in the lipoprotein lipase (*LPL*) gene have been associated with lipid level variability and obesity; however, their role in energy homeostasis remains unclear. The aim of this study was to investigate the association of *LPL* single-nucleotide variants (SNVs) with lipid parameters and leptin concentrations in a cohort of prepubertal children. The sample population comprised 635 boys and 631 girls, with available information on lipid profiles and leptin levels. **Methods:** Five *LPL* SNVs (rs258, rs316, rs326, rs320, and rs328) were genotyped by Real-Time PCR using predesigned TaqMan™ Genotyping Assays. **Results:** An association of the *LPL* SNV rs258 was found with non-esterified fatty acid (NEFA) levels in males and with leptin concentrations in both sexes. On the other hand, an association of the *LPL* SNV rs326 was observed with low-density lipoprotein cholesterol (LDL-C) and apolipoprotein B (Apo-B) levels, displaying opposite trends in males and females. No significant associations with any of the parameters under study were observed for the remaining *LPL* SNVs. **Conclusions:** These results suggest that functional differences among *LPL* SNVs may either be related to an enhancement of catalytic activity or modulation of lipoprotein binding affinity, influencing the efficiency of remnant lipoprotein clearance.

## 1. Introduction

The lipoprotein lipase (LPL) enzyme plays a central role in lipid metabolism, catalyzing the hydrolysis of triglycerides (TGs) from circulating chylomicrons and very-low-density lipoproteins (VLDLs) into non-esterified fatty acids (NEFAs) and glycerol. NEFAs are then taken up by peripheral tissues for energy production, or they are stored in adipose tissue as TGs, which increases fat mass [1,2,3]. Fat mass is related to the expression of leptin, a key adipokine that exerts both direct and indirect effects on adipocyte metabolism [4,5]. In this sense, LPL appears to be a key regulator of both lipid metabolism and energy balance [1,3,6].

Adipokines secreted by mature adipocytes act as hormonal signals for energy balance and adiposity. Among these, leptin is a pivotal regulator of energy homeostasis and obesity [7] but also plays a prominent role in regulating lipid metabolism by stimulating lipolysis and fatty acid oxidation while downregulating lipogenesis [5,8,9]. Leptin is not only influenced by fat mass but also by adipocyte function and differentiation state, processes in which *LPL* expression plays a regulatory role [1,6]. Animal studies have demonstrated that central leptin infusion downregulates *LPL* gene expression, specifically in visceral adipose depots, suggesting that leptin may redirect lipid partitioning away from storage pathways [10].

The *LPL* gene, located on chromosome 8p22 and comprising 10 exons, is highly polymorphic [11], and the relationship between *LPL* single-nucleotide variants (SNVs) and lipid profiles has been extensively studied [12,13,14,15,16]. In addition, although studies regarding adipokines are scarce, some studies have also reported links between certain variants such as *LPL* SNV rs320 and rs328 and circulating levels of leptin and other adipokines, both in healthy children [17] and in pediatric obesity settings [18].

The study of how *LPL* variants influence both lipid profiles and leptin levels may help clarify the molecular connections between these two key molecules in lipid metabolism and energy homeostasis. This cross-sectional study aims to investigate the association of common *LPL* gene variants with lipid parameters and circulating leptin levels in a cohort of healthy prepubertal children.

## 2. Materials and Methods

### 2.1. Subjects

The studied cohort population comprised 1266 Spanish Caucasian children (635 males and 631 females) aged 6–8 years. This analysis is a sub-study within the comprehensive Four Provinces Study (4P), a cross-sectional examination of cardiovascular risk factors in Spain [19]. All participants were confirmed to be free of pre-existing endocrine, metabolic, hepatic, or renal disorders.

The study protocol adhered to the guidelines of the Helsinki Declaration and complied with the Spanish legal provisions governing clinical research involving human subjects. The study was approved by the Jiménez Díaz Foundation Clinical Research Ethics Committee in Madrid, Spain (approval reference: PIC105-2023 FJD, 15 September 2023). Written informed consent was obtained from parents or legal guardians for their children’s participation in the study.

### 2.2. Anthropometric Measurements

Anthropometric data were obtained following standardized measurement protocols. Participants were evaluated barefoot and wearing light clothing. Body weight was measured with a calibrated digital scale (precision ± 0.1 kg), while height was measured using a portable stadiometer with an accuracy of 0.1 cm. Body mass index (BMI) was then calculated as weight in kilograms divided by the square of height in meters (kg/m^2^). BMI standard deviation scores (BMI SDS) were calculated based on Spanish reference data [20].

### 2.3. Biochemical Data

After a 12-h overnight fast, venous blood samples were collected from each child early in the morning via venipuncture. Following centrifugation, the fractions were separated and immediately stored at −70 °C to ensure preservation for subsequent biochemical and genetic determinations.

Total cholesterol (TC) and TG levels were determined enzymatically using a Technicon RA-1000 Autoanalyzer (Menarini Diagnostics, Naples, Italy). High-density lipoprotein cholesterol (HDL-C) was measured using an RA-1000 analyzer after the precipitation of Apo-B-containing lipoproteins with phosphotungstic acid and Mg^2+^ (Boehringer Mannheim, Mannheim, Germany). Low-density lipoprotein cholesterol (LDL-C) levels were calculated using Friedewald’s formula. Plasma apolipoprotein AI (Apo-AI) and apolipoprotein B (Apo-B) concentrations were quantified by immunoephelometry (Dade Behring, Marburg, Germany). The intra-assay coefficients of variation (CVs) for the main analytes were cholesterol, 1.4%; triglyceride, 1.7%; Apo AI, 1.6%; and Apo B, 4.8%. NEFA levels were measured using the Wako NEFA-C kit (Wako Industries, Osaka, Japan). Leptin levels were measured by ELISA using a commercial kit (Leptin EIA-2395, DRG, Marburg, Germany).

### 2.4. LPL SNVs Genotyping

The selected *LPL* SNVs (rs258, rs316, rs326, rs320, rs328), were genotyped by Real-Time PCR, using predesigned TaqMan™ SNV Genotyping Assays from Thermo Fisher Scientific (Waltham, MA, USA); (C__1842994_10, C__12104227_10, C__1843005_20, C__1843003_10, C__901792_1_, respectively). A QuantStudio3^®^ Real-Time PCR System (Applied Biosystems (Waltham, MA, USA), Thermo Fisher Scientific) was used for allelic discrimination. qPCR was performed using a mixture containing 10 ng of genomic DNA and TaqMan™ Master Mix (Thermo Fisher Scientific, Waltham, MA, USA). Samples were cycled under the recommended conditions: 95 °C for 10 min, 95 °C for 15 s, and 60 °C for 1 min., repeated over 40 cycles.

### 2.5. Statistical Analysis

Statistical analyses were carried out using the SPSS software package, version 25.0 (IBM, New York, NY, USA) and GraphPad Prism version 8 statistical software (San Diego, CA, USA). Descriptive statistics are presented as mean ± standard deviation (SD).

Normality for all quantitative variables was assessed using the Kolmogorov–Smirnov test. Variables with a skewed distribution were logarithmically transformed prior to statistical analysis. Differences in means between males and females were tested using Student’s *t*-test. Genotypic distributions between groups were compared using the χ^2^ test. Analysis of variance (ANOVA) was used to compare the quantitative variables across genotypes. Post hoc comparisons between genotype groups were conducted using Tukey’s test whenever statistically significant differences were detected (*p* < 0.05). Univariate analyses were performed to analyze the differences across genotypes, adjusting for body mass index (BMI) as a continuous covariate.

## 3. Results

The study population consisted of 1266 prepubertal children (635 males and 631 females) with a mean age of 7.2 ± 0.6 years and a BMI of 17.0 ± 2.5 kg/m^2^. Neither BMI nor BMI Z-score differed significantly between sexes (Table 1). Females showed higher levels of leptin (6.1 ± 6.4 ng/mL), compared to males (4.1 ± 5.1 ng/mL) (*p* < 0.001) (Table 1).

### 3.1. Genotype and Allele Frequencies of LPL SNVs

Five SNVs located in the *LPL* gene (rs258, rs316, rs320, rs326, and rs328) were genotyped in the study population. The minor allele frequencies (MAFs) of each SNV were 47.0%, 12.0%, 34.0%, 30.0%, and 14.0%, respectively (Table 2). Genotyped distributions for all *LPL* SNVs were in Hardy–Weinberg equilibrium (HWE) (Table 2).

### 3.2. Association Between LPL SNVs and Biochemical Variables

The associations between the five *LPL* SNVs, BMI, leptin, and lipid profiles were evaluated. Significant relationships were detected for specific lipid parameters in connection with *LPL* SNVs rs258 and rs326.

**Figure 1:**
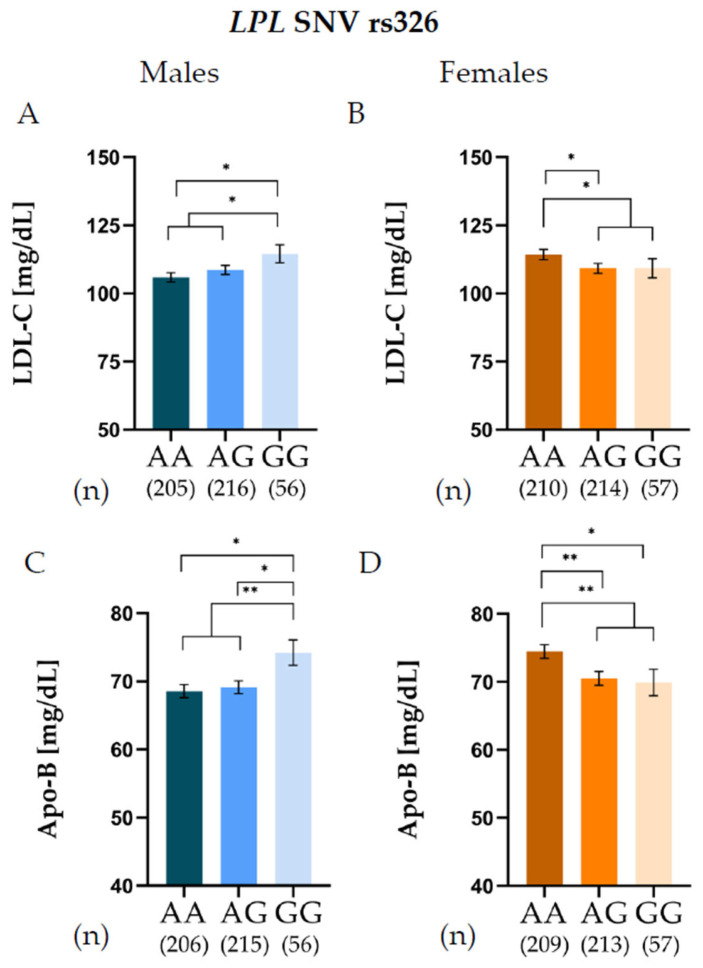
Low-density lipoprotein cholesterol (LDL-C) levels of *LPL* rs326 genotypes in males (**A**) and females (**B**), and apolipoprotein B (Apo-B) levels of *LPL* rs326 genotypes in males (**C**) and females (**D**), adjusted by body mass index (BMI). * *p* < 0.05, ** *p* < 0.01.

For the *LPL* SNV rs326, opposite genotype-phenotype patterns were observed between sexes (Figure 1). In males, G allele carriers exhibited significantly higher LDL-C levels than A allele carriers (Figure 1A), whereas in females the direction of the association was reversed, with G allele carriers showing significantly lower LDL-C concentrations than A allele carriers (Figure 1B). A similar sex-specific pattern was observed for Apo-B levels. Among males, G allele carriers displayed higher Apo-B levels than A allele carriers (Figure 1C), whereas in females, G allele carriers exhibited lower Apo-B concentrations than A allele carriers (Figure 1D). Both associations remained significant after adjusting for BMI (Supplementary Table S1). Similar results were found by analyzing the relationship between the *LPL* SNV rs316 and LDL-C, adjusting for BMI (Supplementary Table S2).

A formal genotype x sex interaction analysis revealed statistically significant associations between the interaction term *LPL* SNV rs326 × sex and LDL-C (*p* = 0.019) as well as Apo-B levels (*p* = 0.003). In addition, a significant interaction was observed between *LPL* SNV rs316 × sex and LDL-C levels (*p* = 0.018).

**Figure 2:**
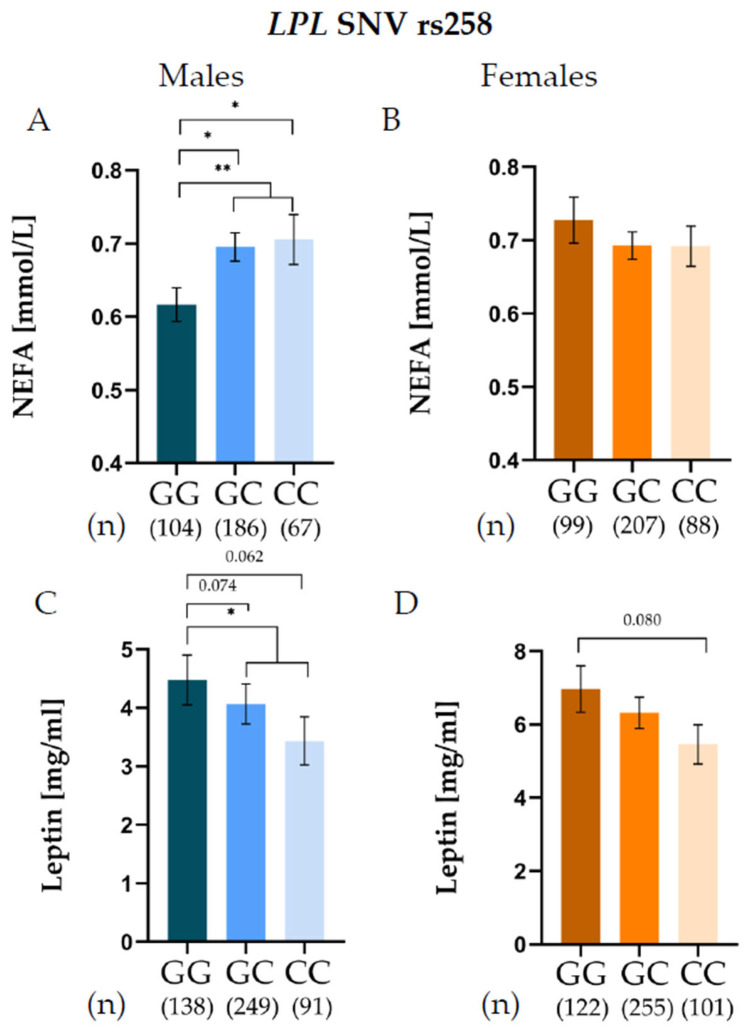
Non-esterified fatty acid (NEFA) levels of *LPL* rs258 genotypes in males (**A**) and females (**B**), and leptin levels of *LPL* rs258 genotypes in males (**C**) and females (**D**). * *p* < 0.05, ** *p* < 0.01.

For the *LPL* SNV rs258 (Supplementary Table S3), male carriers of the C allele exhibited significantly higher NEFA levels compared with GG carriers (Figure 2A), whereas no significant associations were observed in females (Figure 2B). Rs258 was also associated with leptin levels, observing an association of this *LPL* SNV with leptin levels in both males and females: G allele carriers displayed higher leptin concentrations than C allele carriers in males (Figure 2C), and the same trend was observed among females (Figure 2D).

No association of other *LPL* SNVs with any of the parameters studied was observed in this cohort of prepubertal children.

**Figure 3:**
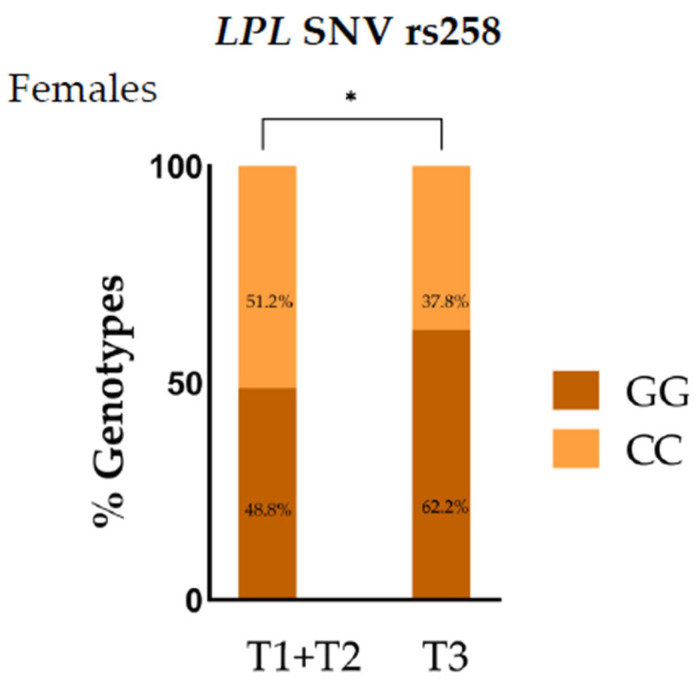
Genotypic distribution of *LPL* SNV rs258 across leptin tertiles in prepubertal females. T1, tertile 1; T2, tertile 2; T3 tertile 3. * *p* < 0.05.

To further analyze this association between *LPL* SNVs and leptin levels, we examined the *LPL* rs258 genotypic distributions among leptin tertiles (T). In females, the prevalence of the GG genotype for *LPL* SNV rs258 was higher in the third tertile (T3) than in the first two tertiles (T1 + T2) (*p* = 0.045) (Figure 3), indicating that GG carriers were associated with higher leptin concentrations.

## 4. Discussion

LPL is a multifunctional enzyme that, in addition to playing an important role in normal lipoprotein metabolism [1,2,3,21], contributes to aspects relating to energy balance and body weight regulation [1,3]. *LPL* genetic variants have been consistently associated with variations in lipid levels [12,13,14,15,16] and influence circulating levels of hormones, such as adipokines [17,18]. In the present study, we analyzed the relationship of circulating lipids and leptin levels with several *LPL* SNVs in a cohort of prepubertal children. The selected variants included those previously reported to be associated with BMI or lipid profile variations [13,14,15,17], as well as highly prevalent *LPL* SNVs that could potentially exert a significant impact on the metabolic parameters assessed.

An association of the *LPL* SNV rs258 with leptin levels and with NEFA in males was observed. Conversely, the *LPL* SNV rs326 showed sex-specific associations with LDL-C and Apo-B levels. Male GG carriers presented significantly higher LDL-C and Apo-B concentrations, whereas female GG carriers exhibited significantly lower levels of LDL-C and Apo-B. Additionally, the *LPL* SNV rs316 showed sex-specific associations with LDL-C, although its relationship was weaker; moreover, we confirmed that rs316 is in linkage disequilibrium with the *LPL* SNV rs326 in the study population. These sex-specific effects observed for the *LPL* variants may reflect early regulatory differences between males and females, even before puberty [22,23]. *LPL* activity is fundamentally regulated by sex hormones, confirming the influence of sex on substrate partitioning [1,23]. Evidence from adolescent cohorts indicated that the effects of the rs328 variant on TGs and HDL-C levels are largely observed in males, highlighting a sex-specific metabolic response [22,23,24].

Several studies have reported associations between *LPL* SNVs and TGs or HDL-C levels; however, few have described a relationship with LDL-C and Apo-B. Momin et al. [16] reported that G allele carriers of rs328 were associated with significantly lower LDL-C levels in patients with type 2 diabetes than in those with CC genotypes. Studies examining the relationship between *LPL* variants and apolipoproteins are scarce. In this context, the association observed between rs326 and LDL-C, and Apo-B levels adds to this limited evidence [22,25].

Rs328 and rs320 are the most extensively studied variants and have been shown to be in strong linkage disequilibrium in European populations [26]. The minor G allele has been linked to an anti-atherogenic profile marked by lower TGs and higher HDL-C levels [17,24,27,28,29]; however, longitudinal data from the Bogalusa Heart Study suggest that these protective effects are modest in childhood and become more pronounced in adulthood, suggesting that the metabolic influence of *LPL* SNVs increases with age [12]. This would justify why no significant associations between lipid parameters and rs320 or rs328 were observed in this prepubertal cohort.

Few studies have examined associations for the *LPL* SNVs rs258 and rs326. Regarding rs326, Tang et al. [13] reported that the minor G allele is associated with lower TGs and higher HDL-C levels and suggested that aging may modulate the effect of *LPL* variants on lipid parameters [13].

The distinct associations observed for *LPL* SNVs suggest the hypothesis that their intronic positions differentially affect LPL function, influencing either its catalytic activity or structural interactions. Rs258 is positioned in intron 5 within the N-terminal domain (exons 2–6), which is crucial for LPL catalytic activity. These non-coding region flank sequences are required for binding the cofactor Apolipoprotein C-II (Apo-CII), which activates LPL catalytic activity [21,30,31]. Variations in this intronic region may modulate LPL function by altering gene expression levels or by subtly affecting Apo-CII binding, thereby influencing lipolysis and circulating NEFA and TGs levels [30]. The observed effect, marked by lower VLDL and TG concentrations, suggests that rs258 could enhance LPL catalytic activity, increasing lipolysis [30]. On the other hand, rs326 resides in intron 8, flanking the C-terminal domain (exons 7–9), which is vital for lipoprotein anchoring, heparin binding, dimerization, and recognition of triglyceride-rich lipoprotein remnants [22]. This structural function provides a plausible explanation for the association of rs326 with LDL-C and Apo-B levels, likely through altered lipoprotein binding affinity [30]. The rs326 variant may affect the turnover or clearance rate of Apo-B-containing particles, linking it primarily to structural lipoprotein regulation rather than enzymatic hydrolysis.

To the best of our knowledge, the analysis of these *LPL* SNVs with leptin levels has been studied only in two studies in children [17,18]. Both rs320 and rs328 have been associated with leptin in a sex-dependent manner. The major T allele of rs320 has been linked to higher circulating leptin concentrations [17]. For rs328, the effect on leptin also differs by sex, with obese males carrying the CC genotype exhibiting higher leptin levels than females, suggesting that this variant may contribute to sex-specific mechanisms in the early pathogenesis of obesity [18].

A major strength of this study is the analysis of a well-characterized, homogeneous cohort of 6-to-8-year-old Caucasian children, minimizing the variability in age, prepubertal influence, and genetic background. Additionally, as participants were prepubertal, potential confounding effects of hormonal changes on the relationship between *LPL* SNVs and the measured metabolic parameters were reduced.

This study has several limitations. First, the absence of data on circulating LPL enzyme levels, LPL expression or functional activity of *LPL* SNVs limits the biological interpretation of the observed associations. Second, multiple genotype–phenotype analyses were conducted across several SNVs and metabolic outcomes without applying a global correction for multiple testing; therefore, the reported associations should be interpreted with caution and considered exploratory and hypothesis-generating, requiring confirmation in independent replication cohorts. In addition, the lack of information on fat mass or waist-related measures prevented their inclusion as covariates in the analyses. Finally, given its observational design, this study cannot establish causal relationships, and the restriction to a single ethnic group limits the generalizability of the findings.

In summary, this study in a prepubertal cohort highlights the differential associations of *LPL* SNVs, rs258 with NEFA and leptin levels, and rs326 with Apo-B metabolism. These findings suggest that *LPL* variants may differ functionally, either by enhancing catalytic activity (lipolytic) or by modulating lipoprotein binding affinity, thereby affecting remnant lipoprotein clearance. These results underscore the need for future studies to further validate the functional effects of these *LPL* SNVs.

## Figures and Tables

**Table 1:** Anthropometric and biochemical characteristics (mean ± SD) by sex.

	6-to-8-Year-Olds (*n* = 1266)		*p*-Value
	Males (*n* = 635)	Females (*n* = 631)	
Age (years)	7.2 ± 0.6	7.2 ± 0.6	ns
BMI (kg/m^2^)	16.9 ± 2.4	17.0 ± 2.5	ns
Z-score BMI	0.02 ± 1.0	0.05 ± 1.0	ns
TC (mg/dL)	181.6 ± 25.8	183.8 ± 28.3	ns
TG (mg/dL)	70.8 ± 23.0	73.9 ± 25.6	*
LDL-C (mg/dL)	107.2 ± 24.8	110.4 ± 26.6	*
Apo-B (mg/dL)	68.8 ± 14.1	71.4 ± 14.9	**
HDL-C (mg/dL)	60.2 ± 13.1	58.8 ± 13.3	ns
Apo-AI (mg/dL)	138.2 ± 19.0	135.6 ± 18.9	*
NEFA (mmol/L)	0.7 ± 0.3	0.7 ± 0.3	ns
Leptin (ng/mL)	4.1 ± 5.1	6.1 ± 6.4	***

ns: non-significant, * *p* < 0.05, ** *p* < 0.01, *** *p* < 0.001.

**Table 2:** Description, genotype, and allelic distribution of the studied single-nucleotide variants (SNVs) in the lipoprotein lipase (*LPL*) gene.

*LPL* SNVs ^a^	Genotype % (*n*)	Allele (%)	HWE *p*-Value
**rs258**	GG 26.8 (304)	G (0.53)	0.0954
g.19954741 G>C	CG 52.3 (593)	C (0.47)	
Intron 5	CC 20.9 (237)		
**rs316**	CC 78.0 (958)	C (0.88)	0.5832
g.19960925 C>A	CA 20.4 (251)	A (0.12)	
Exon 8	AA 1.6 (19)		
**rs326**	AA 43.1 (527)	A (0.66)	0.8218
g.19961928 A>G	AG 45.3 (555)	G (0.34)	
Intron 8	GG 11.6 (142)		
**rs320**	TT 48.9 (600)	T (0.70)	0.9740
g. 19961566 T>G	GT 42.0 (515)	G (0.30)	
Intron 8	GG 9.1 (111)		
**rs328**	CC 74.7 (912)	C (0.86)	0.8705
g. 19962213 C>G	CG 23.3 (284)	G (0.14)	
Exon 9	GG 2.0 (23)		

*LPL*, chr. 8 (8p22); Locus NC_000008.11. HWE Hardy–Weinberg equilibrium. ^a^ Human reference genome GRCh38 (Hg38); genome browse (https://www.ncbi.nlm.nih.gov/ accessed on 16 December 2025).

## Data Availability

The datasets analyzed during the current study are available from the corresponding author upon reasonable request and with permission from the Jiménez Díaz Foundation Clinical Research Ethics Committee.

## Supplementary Materials

The following supporting information can be downloaded at: https://www.mdpi.com/article/10.3390/jcm15020493/s1.

## Abbreviations

The following abbreviations are used in this manuscript:

Apo-AI	Apolipoprotein-AI
Apo-B	Apolipoprotein-B
BMI	Body Mass Index
HWE	Hardy–Weinberg Equilibrium
HDL-C	High-Density Lipoprotein Cholesterol
LDL-C	Low-Density Lipoprotein Cholesterol
MAF	Minor Allele Frequency
NEFA	Non-Esterified Fatty Acid
SNV	Single-Nucleotide Variant
TC	Total Cholesterol
TG	Triglyceride
