# Supplementary material for: Lipoprotein Lipase Genetic Variants rs258 and rs326 Differentially Affect Lipid Profiles and Leptin Levels in Prepubertal Spanish Caucasian Children
Source: J Clin Med. 2026 Jan 8;15(2):493. doi: 10.3390/jcm15020493 (PMC12841871; doi:10.3390/jcm15020493)
Supplement: Supplementary file 1 [file jcm-15-00493-s001.zip › jcm-4078205-supplementary.pdf]

Supplementary Table S1. Association of *LPL* rs326 genotypes with body mass index (BMI), lipid profiles, and leptin levels stratified by sex, adjusted by BMI (mean ± SEM).

rs326 Genotype	Males (N=610)				Females (N=597)			
	**AA (N=257)**	**AG (N=281)**	**GG (N=71)**	**p-value**	**AA (N=263)**	**AG (N=266)**	**GG (N=68)**	**p-value**
BMI (kg/m²)	16.78±0.2	17.09±0.2	17.07±0.3	ns	16.87±0.2	17.15±0.2	16.98±0.3	ns
TC (mg/dl)	179.61±1.7	182.50±1.4	185.93±3.0	ns	185.58±1.8	183.05±1.7	182.23±3.3	ns
TG (mg/dl)	71.06±1.5	70.23±1.3	69.43±2.9	ns	74.70±1.8	71.90±1.3	72.62±2.6	ns
HDL-C (mg/dl)	60.09±0.8	60.38±0.8	59.28±1.7	ns	58.89±0.9	59.46±0.8	59.62±1.4	ns
LDL-C (mg/dl)	105.19±1.7	108.09±1.4	112.77±3.1	AA+AG vs. GG (0.038)<br>AA vs. GG (0.034)	111.75±1.7	109.2±1.6	108.62±3.1	AA vs. AG+GG (0.039)<br>AA vs. AG (0.058)
Apo-AI (mg/dl)	137.26±1.1	138.47±1.2	138.71±2.1	ns	136.83±1.19	135.82±1.13	135.45±2.26	ns
Apo-B (mg/dl)	67.96±0.9	68.94±0.8	73.09±1.7	AA+AG vs. GG (0.007)<br>AA vs. GG (0.014)<br>AG vs. GG (0.010)	73.07±1.0	70.67±0.9	69.58±1.8	AA vs. AG+GG (0.003)<br>AA vs. GG (0.046)<br>AA vs. AG (0.008)
NEFA (mmol/L)	0.67±0.02	0.69±0.02	0.70±0.04	ns	0.72±0.02	0.71±0.02	0.72±0.04	ns
Leptin (ng/ml)	3.97±0.3	4.23±0.3	4.14±0.7	ns	6.30±0.5	6.28±0.4	5.36±0.6	ns

Supplementary Table S2. Association of *LPL* rs316 genotypes with body mass index (BMI), lipid profiles, and leptin levels stratified by sex, adjusted by BMI (mean ± SEM).

rs316 Genotype	Males (N=611)				Females (N=599)			
	CC (N=471)	CA (N=126)	AA (N=14)	p-value	CC (N=475)	CA (N=120)	AA (N=4)	p-value
BMI (kg/m²)	16.93±0.1	17.08±0.3	16.84±0.6	ns	17.03±0.1	16.90±0.3	18.29±1.0	ns
TC (mg/dl)	180.73±1.2	185.41±2.4	183.33±3.7	ns	184.39±1.3	181.71±2.5	211.03±4.3	ns
TG (mg/dl)	70.91±1.1	68.71±1.8	74.54±10.0	ns	73.37±1.2	73.06±1.8	67.20±8.6	ns
HDL-C (mg/dl)	60.36±0.6	59.72±1.1	56.00±3.1	ns	59.10±0.6	59.34±1.1	65.18±6.2	ns
LDL-C (mg/dl)	106.15±1.1	111.96±2.3	112.44±3.4	CC vs. CA+AA (0.002)<br>CC vs. CA (0.004)	110.71±1.2	107.76±2.3	132.41±0.9	CA vs. AA (0.056)
Apo-AI (mg/dl)	138.02±0.9	138.56±1.6	133.86±5.3	ns	135.78±0.9	137.19±1.6	150.00±12.3	ns
Apo-B (mg/dl)	68.53±0.6	70.62±1.3	72.06±2.5	CC vs. CA+AA (0.021)<br>CC vs. CA (0.031)	71.94±0.7	69.72±1.3	84.80±6.6	CA vs. AA (0.038)
NEFA (mmol/L)	0.68±0.02	0.68±0.03	0.61±0.11	ns	0.72±0.02	0.69±0.03	0.97±0.3	ns
Leptin (ng/ml)	4.07±0.2	4.35±0.5	3.38±1.1	ns	6.09±0.3	6.40±0.8	9.27±3.1	ns

Supplementary Table S3. Association of *LPL* rs258 genotypes with body mass index (BMI), lipid profiles, and leptin levels stratified by sex (mean ± SEM).

rs258 Genotype	Males (N=557)				Females (N=560)			
	GG (N=159)	GC (N=287)	CC (N=111)	p-value	GG (N=139)	GC (N=301)	CC (N=120)	p-value
BMI (kg/m²)	17.16±0.2	16.92±0.2	16.67±0.2	ns	17.38±0.3	16.93±0.2	16.94±0.3	ns
TC (mg/dl)	180.60±2.2	181.74±1.5	181.54±2.1	ns	187.63±2.5	182.08±1.7	184.07±2.3	ns
TG (mg/dl)	72.02±2.1	71.46±1.3	70.51±2.2	ns	76.64±2.6	73.89±1.3	72.46±2.1	ns
HDL-C (mg/dl)	59.73±1.0	59.73±0.8	61.64±1.3	ns	59.06±1.2	59.05±0.7	59.20±1.1	ns
LDL-C (mg/dl)	106.86±2.1	107.71±1.5	105.80±2.1	ns	113.24±2.5	108.26±1.5	110.68±2.2	ns
Apo-AI (mg/dl)	138.27±1.5	139.04±1.1	137.50±1.7	ns	138.22±1.6	136.15±1.1	135.93±1.7	ns
Apo-B (mg/dl)	68.93±1.2	69.10±0.8	69.97±1.2	ns	73.80±1.4	70.99±0.8	72.05±1.3	ns
NEFA (mmol/L)	0.62±0.02	0.70±0.02	0.71±0.03	GG vs. CC (0.027)<br>GG vs. GC (0.012)<br>GG vs. GC+CC (0.007)	0.73±0.03	0.69±0.02	0.69±0.03	ns
Leptin (ng/ml)	4.48±0.4	4.07±0.3	3.44±0.4	GC vs. CC (0.074)<br>GG vs. CC (0.062)<br>GG vs. GC+CC (0.041)	6.97±0.6	6.32±0.4	5.46±0.5	GG vs. CC (0.080)

Supplementary Table S4. Association of *LPL* rs320 genotypes with body mass index (BMI), lipid profiles, and leptin levels stratified by sex (mean ± SEM).

**rs320 Genotype**	**Males (N=591)**				**Females (N=586)**			
	**TT (N=307)**	**TG (N=233)**	**GG (N=51)**	**p-value**	**TT (N=269)**	**TG (N=258)**	**GG (N=59)**	**p-value**
BMI (kg/m^2)	16.71±0.2	17.02±0.2	17.22±0.3	ns	16.88±0.2	17.19±0.2	16.85±0.4	ns
TC (mg/dl)	181.74±1.4	181.09±1.7	181.68±3.6	ns	181.96±1.7	184.26±1.8	188.16±3.3	ns
TG (mg/dl)	71.65±1.4	69.52±1.4	66.37±2.2	ns	71.02±1.4	74.27±1.4	79.57±5.3	ns
HDL-C (mg/dl)	60.20±0.9	59.79±0.9	61.09±1.6	ns	58.76±0.8	58.72±0.8	58.32±0.8	ns
LDL-C (mg/dl)	107.21±1.4	107.32±1.6	107.32±3.5	ns	108.99±1.7	110.85±1.7	113.93±3.4	ns
Apo-AI (mg/dl)	138.03±1.0	137.03±1.3	142.55±2.7	ns	135.59±1.2	134.95±1.2	135.27±2.1	ns
Apo-B (mg/dl)	69.22±0.8	68.26±0.9	68.95±2.2	ns	70.80±0.9	71.56±1.0	74.32±1.7	ns
NEFA (mmol/L)	0.66±0.02	0.70±0.02	0.68±0.04	ns	0.70±0.02	0.72±0.02	0.72±0.05	ns
Leptin (ng/ml)	4.04±0.3	4.17±0.4	3.50±0.7	ns	6.10±0.5	6.36±0.5	5.78±0.8	ns

Supplementary Table S5. Association of *LPL* rs328 genotypes with body mass index (BMI), lipid profiles, and leptin levels stratified by sex (mean ± SEM).

rs328 Genotype	Males (N=585)				Females (N=585)			
	**CC (N=445)**	**CG (N=130)**	**GG (N=10)**	**p-value**	**CC (N=432)**	**CG (N=140)**	**GG (N=13)**	**p-value**
BMI (kg/m²)	16.76±0.1	17.30±0.2	16.80±007	ns	16.96±0.1	17.14±0.3	16.29±0.6	ns
TC (mg/dl)	181.49±1.2	181.83±2.2	185.32±8.9	ns	182.77±1.4	185.45±2.5	198.33±6.9	ns
TG (mg/dl)	70.23±1.1	71.95±1.8	60.49±3.9	ns	72.70±1.2	74.40±1.9	85.47±10.7	ns
HDL-C (mg/dl)	60.37±0.7	58.94±1.0	62.21±3.7	ns	58.68±0.6	59.09±1.3	57.07±2.2	ns
LDL-C (mg/dl)	107.02±1.70	108.49±1.9	111.01±7.9	ns	109.55±1.3	111.78±2.3	124.152±6.3	ns
Apo-AI (mg/dl)	138.15±0.9	137.43±1.7	140.70±5.9	ns	135.68±0.9	134.22±1.6	132.85±4.3	ns
Apo-B (mg/dl)	68.72±0.7	69.12±1.2	72.90±4.6	ns	71.22±0.7	72.32±1.3	76.77±3.3	ns
NEFA (mmol/L)	0.68±0.02	0.72±0.03	0.60±0.05	ns	0.72±0.02	0.69±0.03	0.63±0.08	ns
Leptin (ng/ml)	4.06±0.24	4.18±0.6	2.92±0.8	ns	6.41±0.4	5.39±0.5	6.21±2.0	ns
